# Social Preference Tests in Zebrafish: A Systematic Review

**DOI:** 10.3389/fvets.2020.590057

**Published:** 2021-01-22

**Authors:** Asahi Ogi, Rosario Licitra, Valentina Naef, Maria Marchese, Baldassare Fronte, Angelo Gazzano, Filippo M. Santorelli

**Affiliations:** ^1^Neurobiology and Molecular Medicine, Istituto di Ricovero e Cura a Carattere Scientifico Stella Maris, Pisa, Italy; ^2^Department of Veterinary Sciences, University of Pisa, Pisa, Italy

**Keywords:** zebrafish, social preference test, social behavior, oxytocin, disease model, animal welfare

## Abstract

The use of animal models in biology research continues to be necessary for the development of new technologies and medicines, and therefore crucial for enhancing human and animal health. In this context, the need to ensure the compliance of research with the principles Replacement, Reduction and Refinement (the 3 Rs), which underpin the ethical and human approach to husbandry and experimental design, has become a central issue. The zebrafish (*Danio rerio*) is becoming a widely used model in the field of behavioral neuroscience. In particular, studying zebrafish social preference, by observing how an individual fish interacts with conspecifics, may offer insights into several neuropsychiatric and neurodevelopmental disorders. The main aim of this review is to summarize principal factors affecting zebrafish behavior during social preference tests. We identified three categories of social research using zebrafish: studies carried out in untreated wild-type zebrafish, in pharmacologically treated wild-type zebrafish, and in genetically engineered fish. We suggest guidelines for standardizing social preference testing in the zebrafish model. The main advances gleaned from zebrafish social behavior testing are discussed, together with the relevance of this method to scientific research, including the study of behavioral disorders in humans. The authors stress the importance of adopting an ethical approach that considers the welfare of animals involved in experimental procedures. Ensuring a high standard of animal welfare is not only good for the animals, but also enhances the quality of our science.

## Introduction

The zebrafish (*Danio rerio*) is a small tropical freshwater fish, belonging to the cyprinid family (1). This teleost fish first attracted the attention of aquarists, and about four decades ago began to gain popularity in biomedical research as an animal model (2). Native to the Himalayan region, this species has been found in a wide variety of environments, such as rice crops, ponds, rivers, and streams (3). Surveys carried out in wild populations suggest that zebrafish prefer warm (24–35°C) and slow-flowing waters, characterized by slight alkalinity (pH 7–8), high transparency and the presence of plant coverage (4, 5). Zebrafish are asynchronous, batch spawners. Their reproductive behavior in the wild is dependent upon food availability and positively correlated with increased rainfall (6).

Olfactory signals play an essential role in zebrafish reproduction: the presence of glucuronic steroids, released into the water by males, induces ovulation in females. After ovulation, females release other hormones, which in turn induce the release of gametes by males (7). The fertilized eggs are demersal, transparent, usually hatch in 48–72 h, and do not receive parental care (6). With regard to biometric data, Ribas and Piferrer (8) report that the live weight of adult zebrafish varies between 500 and 900 mg, while their body length, from the head to the point at which the tail forks, ranges from 22 to 38 mm; they also note that the two sexes show somatic dimorphism, the females being larger and heavier than the males. Although the average life span of these fish is about 3 years, it has been shown that in laboratory conditions they can survive for more than 5 years (9).

The zebrafish is omnivorous and its diet consists mainly of zooplankton, phytoplankton and insects (8). In the laboratory, zebrafish are mainly fed a mixture of live prey, such as ciliates, rotifers and *Artemia*, and/or commercial dried feeds. The use of live prey provides behavioral enrichment designed to improve zebrafish welfare by encouraging the animal's natural predatory behavior (10). Such physical enrichment has become mandatory for rodents, but as yet there is no evidence suggesting that it produces improvements in zebrafish welfare (11).

Wild zebrafish live in social groups composed of varying numbers of individuals, depending mainly on the water flow of the site where they live. In still and slow-flowing waters, zebrafish have been found in small groups of 7–10 individuals, while in fast-flowing rivers, they can be found in large shoals of up to 300 fish (12, 13). The typical laboratory housing for zebrafish consists of transparent tanks connected to a flow-through system. The tanks are generally kept without environmental enrichment and their volume ranges from 1 to 10 L (11). The tanks have a water flow rate of about 10 L/h. Graham et al. (12) and Varga (14) suggested a stocking density of ~5 fish per liter, but the Federation of European Laboratory Animal Science Associations (FELASA), in its “housing and husbandry recommendations,” reported that a fish density of between 3 and 12 adult fish/L has no impact on reproductive performance. It has also been suggested that neighboring transparent tanks could act as potential stress reducers (11).

Despite the variability of environmental conditions in nature, a dark/light cycle of 10/14 h is recommended. That said, a 12/12 h cycle does not seem to affect animal well-being (11). Conversely, water quality may affect zebrafish well-being, although the same authors suggest that zebrafish seem adaptable to a wide range of conductivities (150–1,700 μS/cm) and pH values (6–10). Nitrogen compounds are toxic to fish and total ammonia, nitrite and nitrate levels should be kept below 0.1, 0.3, and 25 mg/L, respectively. Although changes in the dark/light cycle or water conditions could be associated with reduced egg production, the zebrafish is adaptable and can survive in a wide range of laboratory conditions (11).

The assessment of welfare in this animal species is based on reproductive success and the absence of signs of illness or excessive stress. Although cortisol levels are a useful marker of stress in rodents, and can also be assessed in fish, either by analyzing the water or as total body content, they do not seem to be a good indicator of fish welfare (15).

The zebrafish are widely used animal models in several research fields, including translational study of human and animal diseases (16–18), developmental biology (19), and pharmacology and toxicology (20, 21).

Since this animal model may represent a valid alternative to the use of higher-order animals, such as mammals, its use complies with article 13 of the EU directive on the protection of animals used for scientific purposes (Directive 2010/63/EU). This directive provides specific guidelines, requiring researchers to choose procedures which “involve animals with the lowest capacity to experience pain, suffering, distress or lasting harm.” Moreover, some authors argue that since the zebrafish is a “lower vertebrate,” its use complies with the “3 Rs” principles (22), extending the replacement concept to less sentient animals in general.

Even though rodents, such as mice (23), rats and prairie voles (24), are the current “go to” models for studying human social disorders, the social activity of zebrafish makes this species a valuable model for behavioral neuroscience research. Furthermore, unlike rodents, zebrafish are predominantly diurnal and less sensitive to environmental disturbances (25), and therefore facilitate behavioral observation. According to Saverino and Gerlai (19), the zebrafish also shows a higher degree of social cohesion compared with rodents. Finally, from a welfare perspective, although both species are social sentient beings, rodents, compared with zebrafish, adapt less easily to laboratory conditions.

Adult zebrafish, both in natural and in laboratory conditions, form groups to maximize their foraging efficiency and avoid predation (26). At 2 weeks of age, young zebrafish start to swim close to each other, forming social groups (27). The group of fish that “remain together for social reasons” is commonly termed a shoal (28), whereas a school is any group of fish within a shoal that exhibits a collective behavior characterized by alignment of bodies and coordinated swimming velocity (28). This schooling behavior has evolutionary advantages, such as more effective predator evasion and increased swimming efficiency (28, 29). Even though there are significant differences between shoaling and schooling, these two terms are often used interchangeably in research studies. Study of the zebrafish behavioral repertoire includes behaviors related to single individuals, such as modalities of swimming (30) and of prey capture (31), emotional responses to stimuli (32), and cognitive abilities (33), as well as group-level behaviors, such as shoaling, schooling (34) and courtship (35).

The two major approaches employed to investigate the diversity of social behavior displayed by animals, including humans, focus either on the mechanism or on the function of the studied behavior (36). To investigate the mechanisms of behaviors, neuroscientists have developed highly specialized tool kits that allow exploration of normal and abnormal social behaviors, particularly in species well-suited to the laboratory (37), such as mice (38) and zebrafish (19, 39). In the laboratory, the study of zebrafish group forming is fundamentally approached in two different ways (40). One is to observe freely moving fish and record various behavioral parameters of a shoal, such as distances between subjects and synchronization of movements within the shoal (41). The second is to conduct a social preference test, which consists of using social stimuli in order to investigate the behavioral response of a single fish to a shoal/s (19).

Additional methods to test social behavior in zebrafish are the mirror biting test and the predator exposure test. The mirror biting test involves measuring the social/aggressive response of a solitary zebrafish to its mirror image (42). The predator exposure test, involving the presence of live or robotic, sympatric or allopatric, predators, induces stress and fear in zebrafish, increasing their shoal cohesion (43); this anti-predatory response has been mostly employed to study anxiety and fear-related behavior (44).

## The Social Preference Test

Social preference can be defined as the predilection of individuals to live near conspecifics (45). In zebrafish, this social behavior is assessed by observing how an individual responds to, or interacts with, a social stimulus. Social preference tests have been developed for zebrafish that are similar to those used in rodents (46). Social preference tests are composed of two operational phases. The first is the habituation phase, during which the tested zebrafish is left alone in a chamber of the test tank to explore the novel environment. The second is the interaction phase, which starts with the introduction of the social stimulus consisting of one, or usually two, small groups of live conspecifics. Alternatively, the social stimulus can consist of a pre-recorded video of live fish shown on a computer monitor, computer animated (moving) images of zebrafish (40), or virtual reality systems (47).

**Figure 1:**
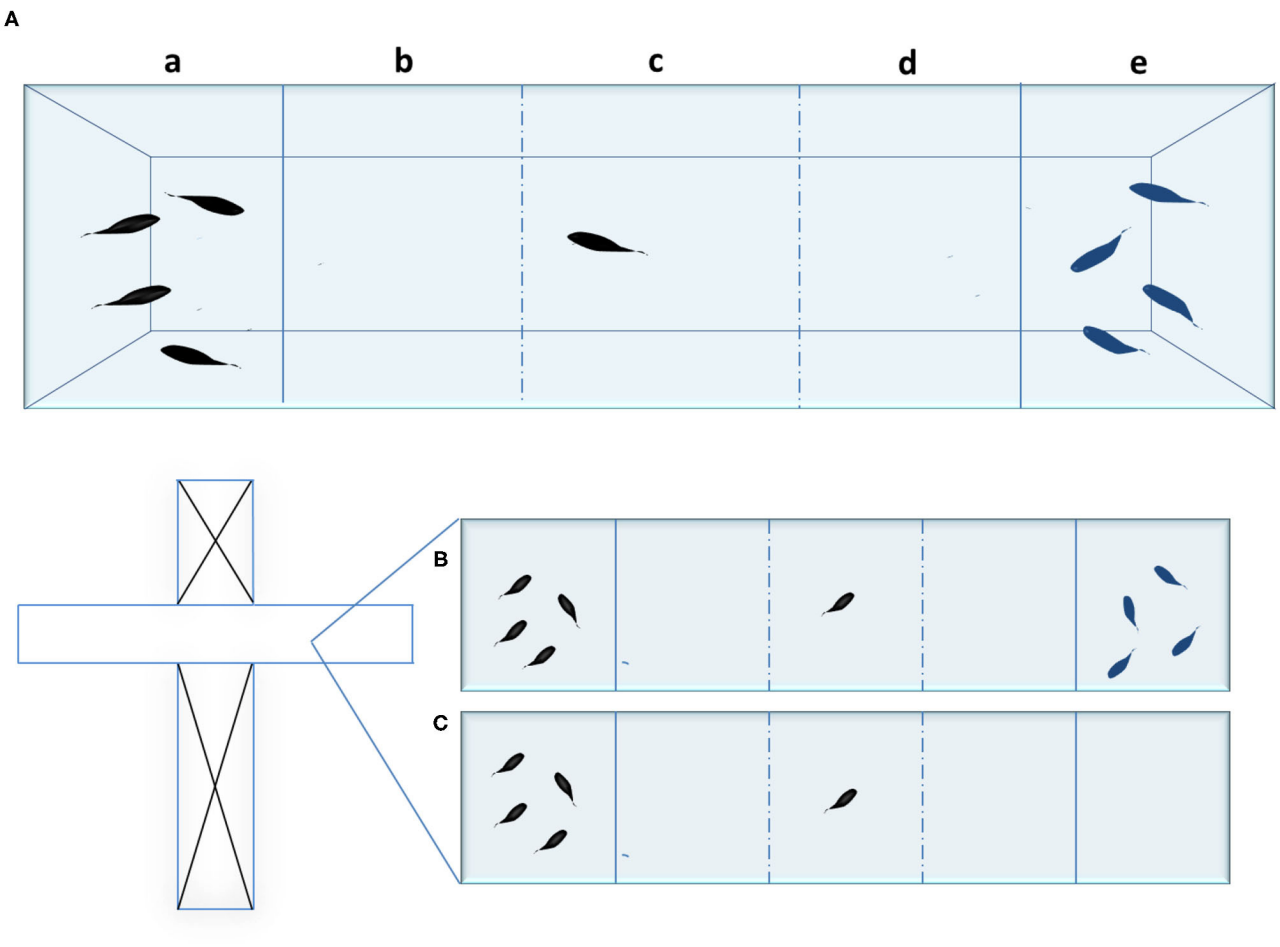
**(A)** social preference test tank with three chambers (five areas) and two social stimuli. a) left social stimulus chamber; b) +c) +d) tested fish chamber; e) right social stimulus chamber; b) area of social preference for the left social stimulus; d) area of social preference for the right social stimulus c) area of no social preference; **(B)** T-maze modified for the social preference test with two social stimuli. **(C)** T-maze modified for the social preference test with one social stimulus. The dashed lines show where additional chamber dividers can potentially be placed.

To test social behavior toward a single stimulus, a rectangular-shaped tank can be used; this is divided into two chambers with a transparent barrier that allows visual contact between the tested fish and the shoal. To test, simultaneously, preference between two different social stimuli, it is common to use a rectangular tank divided into three chambers (Figure 1A). Alternatively, a T-maze, adapted for this same purpose, can be used; in this case, the guillotine doors are used to seal off the horizontal section of the maze, which is then divided into three chambers (Figures 1B,C). The T-maze is a laboratory apparatus commonly used to test cognitive abilities in rodents (48).

During the test, the fish are left to swim and roam free within their respective chambers (26) and the behavioral response of the tested zebrafish is video-recorded with a camera and tracked using specific software (see Supplementary Video). Social preference is usually assessed by measuring the time spent by the observed fish in the areas proximal to the social stimulus; this time is then expressed as a percentage of the total time. The preference area can also be divided into zones of “strong” and “weak” social preference, according to the distance from the social stimulus chamber, as recently reported by Aslanzadeh et al. (49) and Landin et al. (50).

The social preference test could be useful for assessing variables that can influence zebrafish social behavior in laboratory conditions, and for investigating, from a translational perspective, the effects of drugs, medications and hormones. The social preference test may also be useful in efforts to find a valid zebrafish model for human neuropsychiatric and neurodevelopmental disorders characterized by social impairments.

It is well-known that social behavior assessments can be influenced by multiple factors, such as differences in individual phenotypes, life-history stages and social contexts (37). Social preference in zebrafish can be influenced by many endogenous and exogenous variables. These variables may be classified into two categories: the individual characteristics of the observed fish, namely its age, sex and personality, and the characteristics of the group members (i.e., of the social stimulus in the social preference test), namely their number, size, sex ratio, phenotype, and kinship between individuals. On the other hand, the exogenous variables that may potentially affect social preference findings are essentially the different environmental conditions (i.e., the water temperature and the tank volume), the test room brightness, the size and number of the preference areas, the presence of environmental enrichment, and the duration of the habituation and interaction phases.

## Materials and Methods

**Figure 2:**
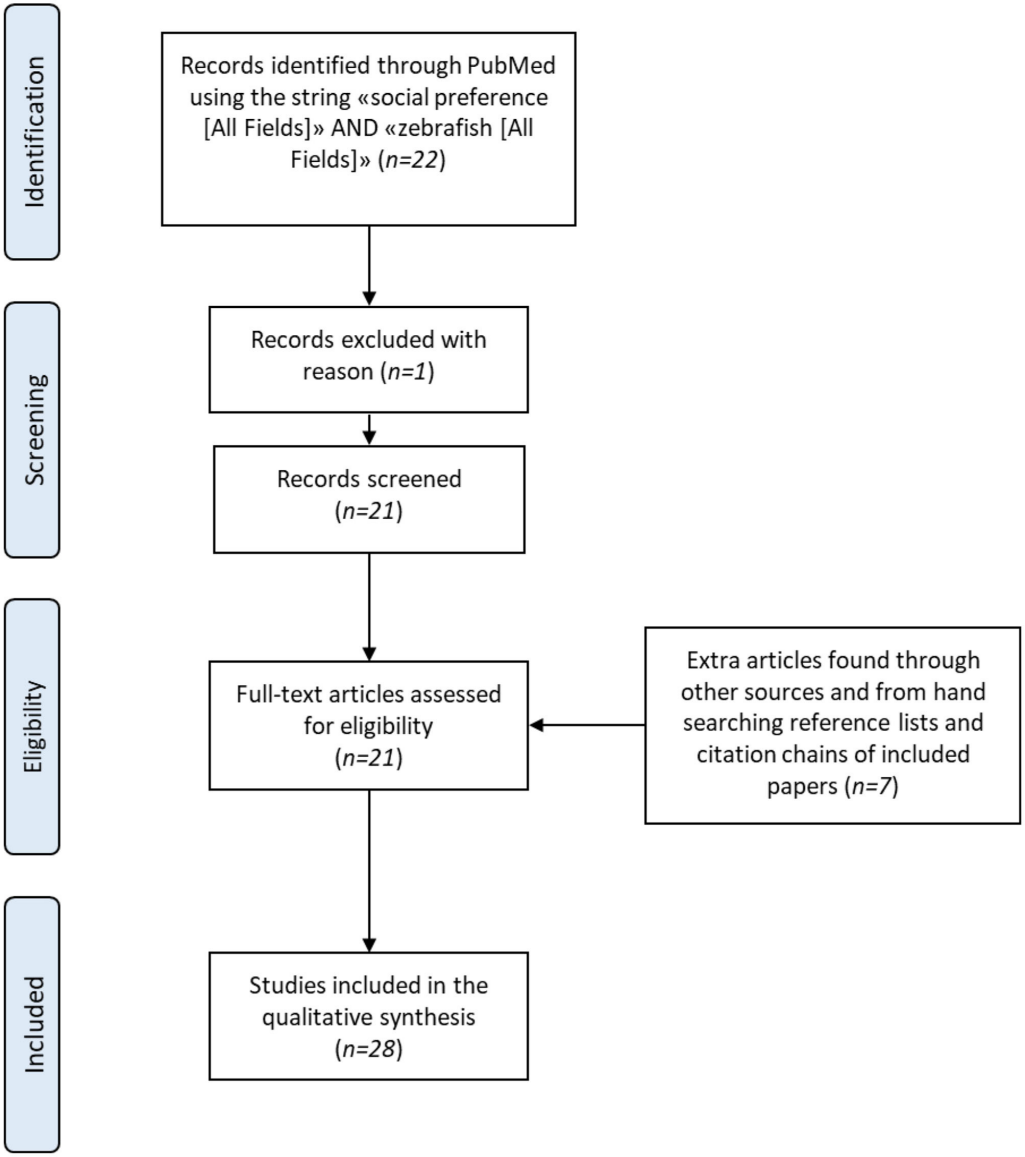
PRISMA flow diagram of the literature search process.

A literature search of the PubMed database up to March 31st, 2020 was performed using the terms “social preference” (All Fields) AND “zebrafish” (All Fields). The search yielded 22 matches, but one article was excluded because it was not an original research paper. The references of these publications were examined and a further 8 papers were identified; these were critically evaluated and one was excluded because the authors tested more than one fish simultaneously, i.e., they combined a shoaling/schooling-test with a social preference test. Overall, 28 articles were included in this review (see Figure 2 for a schematic summary of the methodology) and then grouped into three categories: 4 studies carried out with untreated wild-type (WT) zebrafish, 20 with pharmacologically treated WT zebrafish, and 4 with genetically manipulated fish. Table 1 gives details of the experimental protocols of all the studies included in the review.

**Table 1:** An overview of social preference test in zebrafish.

**Assessed variable**	**Strain**	**Tested fish per group**	**Sex of tested fish**	**Age of tested fish**	**Test tank characteristics**	**Social stimulus**	**Behavioral protocols**	**Results**	**References**
**STUDIES CARRIED OUT IN UNTREATED WT ZEBRAFISH**									
Phenotype and rearing management	AB and *nacre*	33–38	Mixed	5–6 months	One 245 L tank (122 × 32 × 55 cm, L × W × H) divided into three chambers: one for AB, one for n*acre* and a central one for the tested fish	4 AB vs. 4 *nacre* fish	Habituation phase: 10 min; interaction phase: 15 min	Zebrafish showed positive social preference with individuals of the same phenotype with which they were raised	(51)
Visual characteristics of animated zebrafish images	SF	10	50-50% m-f	6–8 months	One 40 L tank (51 × 30 × 25 cm, L × W × H) with flat LCD computer screens for social stimuli on its left and right sides	1 modified vs. 1 unmodified computer-animated female zebrafish	Habituation phase: 10 min; interaction phase: 5 min	Visual characteristics of animated zebrafish images affected social preference	(19)
Body size of conspecifics	n.a.	6–7	Males	3–12 months	One 14 L tank (58 × 15 × 15 cm, L × W × H) divided into three chambers: one for large conspecifics, one for small conspecifics, and a central one for tested fish	3 large vs. 3 small conspecifics	Habituation phase: 10 min; interaction phase: 10 min	Zebrafish males preferred to socialize with larger body size conspecifics compared with smaller ones	(49)
Familiarity of conspecifics	AB	n.a.	n.a.	n.a.	One tank (19 × 13.2 × 9.3 cm, L × W × H) divided into five chambers: one for familiar conspecifics, one for unfamiliar conspecifics, two empty, and a central one for tested fish	3 familiar vs. 3 unfamiliar conspecifics	Habituation phase: n.a.; interaction phase: 5 min	Zebrafish showed increased social preference for unfamiliar over familiar conspecifics	(26)
Temperature increase from 26 to 34°C	AB	15	50-50% m-f	12 months	Three aligned 4 L tanks (25 × 28 × 16 cm, L × W × H). One for the social stimulus, one empty for no social stimuli, and a central one for tested fish	3 conspecifics	Habituation phase: n.a.; interaction phase: 10 min	Heat treatment reduced social preference	(52)
**STUDIES CARRIED OUT IN PHARMACOLOGICALLY TREATED WT ZEBRAFISH**									
Alcohol treatment, chronic (10 days in 0.50% alcohol v/v) and then acute (1 h in 0.25, 0.50, or 1.00% alcohol v/v)	AB and SF	13–18	50-50% m-f	12 months	One 37 L tank (50 × 25 × 30 cm, L × W × H) with flat LCD computer screens, for displaying social stimuli, on its left and right sides	5 computer-animated conspecifics	Habituation phase: 8 min; interaction phase: 8 min	AB fish exposed to chronic and subsequently to 1.00% acute alcohol treatment showed reduced social preference, while SF fish did not	(53)
Lysergic acid diethylamide (LSD) treatment (20 min in 5, 25, 50, 75, 100 or 250 μg/L)	SF	10	Mixed	5–7 months	Five areas of a modified T-maze (50 × 10 × 10 cm, L × W × H). One chamber for the social stimulus, one empty chamber for no social stimuli, and the central chamber (divided into three areas), for tested fish	1 conspecific	Habituation phase: 30 s; interaction phase: 6 min	LSD treatment did not influence zebrafish social preference	(54)
Ketamine treatment (20 min in 2, 20, or 40 mg/L)	SF	12	Mixed	5–7 months	Five areas of a modified T-maze (50 × 10 × 10 cm, L × W × H). One chamber for the social stimulus, one empty for no social stimuli, and the central chamber (divided into three areas), for tested fish	1 conspecific	Habituation phase: 30 s; interaction phase: 6 min	Ketamine treatment did not affect zebrafish social preference	(55)
MK-801 treatment (30 min in 2, 20, or 100 μM)	SF	8	50-50% m-f	6–8 months	Three aligned 2 L tanks (21 × 10 × 10 cm, L × W × H). One for social stimulus, one empty for no social stimuli, and a central one for tested fish	5 conspecifics	Habituation phase: n.a.; interaction phase: 30 min	MK-801, at the highest dose (100 μM), significantly reduced fish social preference	(56)
Isotocin and vasotocin injection (0.001–40 ng/kg body weight)	n.a.	10	50-50% m-f	6–12 months	One tank (122 × 32 × 55 cm, L × W × H) divided into three chambers: one for WT conspecifics, one for *nacre* mutants, and a central one for tested fish	4 conspecifics	Habituation phase: 5 min; interaction phase: 15 min	Both neuropeptides increased social preference in a dose-dependent manner	(57)
D1-receptor antagonist SCH23390 treatment (30 min in 0.1 or 1.0 mg/L)	AB and SF	20	50-50% m-f	4–6 months	One 37 L tank (50 × 25 × 30 cm, L × W × H) with flat LCD computer screens for social stimuli on its left and right sides	5 computer-animated female zebrafish conspecifics	Habituation phase: 8 min; interaction phase: 8 min	In AB fish treated with the high concentration of SCH23390, social preference was impaired. In the SF strain, no significant effects were observed	(27)
Indole alkaloid ibogaine treatment (20 min 10 or 20 mg/L)	SF	15	50-50% m-f	5–8 months	Five chambers of a modified T-maze (50 × 10 × 10 cm, L × W × H). One chamber for social stimulus, one empty for no social stimuli, and the central three chambers for tested fish	1 conspecific	Habituation phase: 30 s; interaction phase: 6 min	Ibogaine treatment did not influence zebrafish social preference	(58)
Injection of isotocin, an isotocin antagonist, or vasotocin, a vasotocin antagonist (10 μg/g body weight)	n.a.	20	100% females	4–5 months	One tank (150 × 50 cm, L × W) divided into three chambers: one for social stimulus, one empty for no social stimuli, and a central one for tested fish	8 conspecifics	Habituation phase: n.a.; interaction phase: 10 min	Vasotocin and its antagonist decreased social preference, while isotocin and its antagonist had no significant effects on social preference	(59)
MK-801 treatment (1 h in 100 μM) or acute alcohol treatment (1 h in 0.125 or 0.50% alcohol v/v)	AB	177 for MK-801, 96 for low and 82 for high alcohol treatments	n.a.	3 weeks	One tank (4 × 3.2 cm, L × W) divided into three chambers: one for social stimulus, one empty for no social stimuli and a central one for tested fish	1 or 3 conspecifics	Habituation phase: 15 min; interaction phase: 15 min	After blocking of NMDA receptors, fish exhibited no social preference. Alcohol treatment significantly reduced social preference only at the higher dose	(60)
Injection of amphetamine derivatives DOB (0.05–2 mg/kg), PMA (0.0005–2 mg/kg), MDMA (0.25–20 mg/kg) or ritanserin (0.025–2.5 mg/kg body weight) in association with the maximal doses of DOB, PMA or MDMA	SF	10	50-50% m-f	6–12 months	One tank (122 × 32 × 55 cm, L × W × H) divided into five areas: the outermost chambers as stimulus areas with pictures of zebrafish as social stimuli, and the central three areas (1 chamber) for tested fish	Pictures of 6 conspecifics (SF vs. *nacre*)	Habituation phase: 5 min; interaction phase: 15 min	Inverted-U shape dose-dependent increase in social preference was observed for DOB, PMA and MDMA treatments	(61)
Fluoxetine (15 min in 50 μg/L) and diazepam (15 min in 16 μg/L)	SF	7–10	50-50% m-f	6 months	Three aligned tanks (30 × 15 × 10 cm, L × W × H). One for social stimulus, one empty for no social stimuli, and a central one for tested fish	15 conspecifics	Habituation phase: 30 s; interaction phase: 10 s	Fluoxetine (15 min in 50 μg/L) and diazepam (15 min in 16 μg/L) decreased social preference	(62)
MK-801 treatment (15 min in 5 μM) and subsequent oxytocin, carbetocin or L-368,899 injection (10 ng/kg body weight)	SF (Tübingen)	16	n.a.	6–8 months	Three aligned tanks (30 × 10 × 15 cm, L × W × H). One for social stimulus, one empty for no social stimuli, and a central one for tested fish	15 conspecifics	Habituation phase: 30 s; interaction phase: 5 min	MK-801 induced a decrease in social preference. Oxytocin and carbetocin re-established this behavior, while L-368,899 did not	(63)
Sodium valproate treatment, chronic (from 1 dpf 7 h per day for 6 days in 20 μM) or acute (at 1 dpf 7 h in 100 μM)	AB	60	n.a.	1 month	One tank divided into two chambers: one for social stimulus and one for tested fish (7 × 4.2 cm, L × W).	6 conspecifics	Habituation phase: 20 min; interaction phase: 30 min	Chronic exposure to sodium valproate significantly impaired social preference, while acute exposure did not	(45)
Acute alcohol treatment (at 1dpf 2 h in 0.01, 0.25, 0.50, or 1.00% alcohol v/v)	SF	8	50-50% m-f	4 months	Three aligned tanks. One for social stimulus, one empty for no social stimuli, and a central one for tested fish.	3 conspecifics	Habituation phase: n.a.; interaction phase: 6 min.	Embryonal exposure to alcohol reduced social preference in a dose dependent manner	(64)
Acute alcohol treatment in association with taurine treatment (1 h in 0.25% alcohol v/v and in 42, 150 and 400 mg/ L of taurine)	SF	10–12	50-50% m-f	4–6 months	One tank (25 × 10 × 15 cm, L × W × H) divided into three chambers: one for social stimulus, one empty for no social stimuli, and a central one for tested fish	4 conspecifics	Habituation phase: 30 s; interaction phase: 1 min	Acute alcohol exposure in association with the highest dose of taurine significantly reduced social preference	(21)
Formalin-inactivated *Aeromonoas hydrophila* bacterin injection (50 μL of 4 × 10^5^)	n.a.	12	Mixed	6 months	One tank (24 × 8 × 20 cm, L × W × H) divided into three chambers: one for social stimulus, one empty for no social stimuli, and a central one for tested fish	15 conspecifics	Habituation phase: n.a.; interaction phase: 1 min	Formalin-inactivated *Aeromonoas hydrophila* bacterin injection reduced social preference	(17)
Untreated tannery effluent exposure (30 days in 0.1 and 0.3% of body biomass)	n.a.	15 in each group	Mixed	6–8 months	Three aligned 5 L tanks (20 × 15 × 20 cm, L × W × H). One for social stimulus, one empty for no social stimuli, and a central one for tested fish	6 conspecifics	Habituation phase: 4 min; interaction phase: 4 min	Untreated tannery effluent at the highest dose reduced social preference	(20)
Alcohol treatment, intermittent acute (20 min per day for 4 days in 1% alcohol v/v) and intermittent chronic (20 min per day for 16 days in 1% alcohol v/v)	SF	25 in each group	Mixed	4–5 months	Three aligned tanks. One for social stimulus, one empty for no social stimuli, and a central one for tested fish	5 conspecifics	Habituation phase: 2 min; interaction phase: 5 min	Acute alcohol exposure significantly reduced social preference, while chronic exposure did not	(65)
Chronic acesulfame treatment (2 months in 1, 10 or 100 mg/L)	AB	12 in each group	n.a.	9 months	One tank (30 × 10 × 10 cm, L × W × H) divided into three chambers: one for social stimulus and two for tested fish	6 conspecifics	Habituation phase: n.a.; interaction phase: 3 min	Chronic acesulfame exposure reduced social preference	(66)
L-368,899 injection (100 μg/g body weight).	AB	21 in each group	50-50% m-f	n.a.	One tank (90 × 30 cm, L × W) divided into three chambers: one for social stimulus, one empty for no social stimuli, and a central one for tested fish	8 conspecifics	Habituation phase: 10 min; interaction phase: 15 min	L-368,899 injection reduced social preference	(50)
L-368,899 treatment (1 h in 100 μM)	AB	77–87 in each group	n.a.	3 weeks	One tank (4 × 3.2 cm, L × W) divided into three chambers: one for social stimulus, one empty for no social stimuli and a central one for tested fish	1 conspecific	Habituation phase: 15 min; interaction phase: 15 min	L-368,899 treatment reduced social preference	(50)
**STUDIES CARRIED OUT IN GENETICALLY ENGINEERED ZEBRAFISH**									
*otpa* and *otpb*		10 in each group	n.a.	n.a.	One tank divided into three chambers: one for social stimulus, one empty for no social stimuli, and one for tested fish	4 conspecifics	Habituation phase: 5 min; interaction phase: 9 min	*otpa* mutants showed reduced social preference as compared with WT and *otpb* mutants	(67)
*reln*		10–12 in each group	Mixed	3–6 months	One tank divided into five chambers: one for social stimulus, three empty, and a central one for tested fish	3 conspecifics	Habituation phase: n.a.; interaction phase: 5 min	*reln* mutants showed similar social preference as compared with WT	(68)
*dyrk1aa*		6–7 in each group	Males	3–12 months	One 14 L tank (58 × 15 × 15 cm, L × W × H) divided into three chambers: one for large conspecifics, one for small conspecifics, and a central one for tested fish	3 large vs. 3 small WT conspecifics	Habituation phase: 10 min; interaction phase: 10 min	*dyrk1aa* heterozygous zebrafish exhibited a significantly higher preference for the large conspecifics, while homozygous KO zebrafish showed no such preference	(49)
*oxtr*		10–14 in each group	Mixed	3–6 months	One tank (20 × 19 × 5 cm, L × W × H) divided into three chambers: one for social stimulus, one empty for no social stimuli, and one for tested fish	4 conspecifics	Habituation phase: 5 min; interaction phase: 10 min	*oxtr* mutants showed normal social preference	(69)

*n.a, data not available; m-f, males-females ratio; SF, short fin; DOB, 2,5-dimetoxy-4-bromo-amphetamine hydrobromide; PMA, para-methoxyamphetamine; MDMA, 3,4 methylene-dioxymethamphetamine; dpf, days post-fertilization*.

## Studies Carried Out With Untreated WT Zebrafish

The purpose of studies of this kind is to observe and describe the variables that can affect zebrafish social behavior in laboratory conditions. The studies in this category examined the influence of phenotype and environmental conditions, as well as the perception of chemosensory cues in WT zebrafish.

An early study of factors affecting zebrafish social preference focused on visual signals, and in particular on fish pigment patterns (51). Engeszer and colleagues reported that zebrafish showed a strong positive social preference for individuals with a similar phenotype: WT preferred WT, and *nacre* (strains with no melanophore stripes) preferred *nacre* (70). Moreover, the authors suggested that visual social preference in zebrafish is not innate, but depends on early environmental conditions, since fish reared in isolation did not show any phenotypic preference, whereas WT raised with *nacre* siblings preferred *nacre*, and *nacre* raised with WT siblings preferred WT (51). The visual characteristics of both living zebrafish and computer-animated zebrafish images could also affect social preference (19). When social stimuli consisted of conspecifics of different sizes, tested zebrafish preferred to interact with fish of larger size, presumably to reduce predation risks (49). In addition, WT AB strain zebrafish of the same age and size and seem to prefer unfamiliar over familiar conspecifics when the separation barrier allows the passage of some water and they are allowed to perceive chemosensory cues (26). Another variable that could influence social preference in zebrafish is water temperature, an abiotic parameter critical for all acquatic animals. Although wild zebrafish have a wide thermal tolerance (from 6.7 to 41.7°C), reduced social preference has been observed as an effect of increasing the water temperature of the rearing tanks from 26 to 34 °C for 21 days (52).

## Studies Carried Out With Pharmacologically Treated WT Zebrafish

The studies in this category were conducted with the aim of identifying the effects of drugs, medications and hormones on social behavior, highlighting their translational implications.

In the last few years, numerous neurobehavioral studies have been carried out to test the effects of different molecules on zebrafish social preference. Results suggest that social preference in adult WT fish may be modulated using the neuropeptides isotocin and vasotocin (homologs of mammalian oxytocin and arginine-vasopressin) and their antagonists (57, 59). This hypothesis has been validated in other freshwater fish species on the basis of evidence that isotocin modulates fish responsiveness to social stimuli (71), in line with what has been observed with oxytocin in mammals (72, 73). In both larvae and adult zebrafish, social preference was abolished after administration of the glutamate antagonist MK-801, which worked by blocking the N-methyl-D-aspartate receptors (56, 60, 63). Treatments with zebrafish “oxytocin” (zOT) or with carbetocin, a zOT receptor agonist, reversed social deficits in zebrafish induced by exposure to the antagonist MK-801, while the zOT receptor antagonist L-368,899 did not reverse the same deficits (63). Administration of L-368,899 was found to inhibit social preference in adult and larval zebrafish, strengthening the evidence that zOT receptors are involved in the regulation of social behavior (50).

Scerbina et al. (27) suggested that genetic differences, too, can affect both behavioral and neurochemical responses in WT zebrafish. In a condition characterized by impaired dopamine-regulated motivation and/or reward mechanisms, induced experimentally through treatment with a dopamine receptor antagonist (SCH23390), WT AB strain fish showed reduced social preference compared with untreated fish of the same strain. On the contrary, the WT short fin (SF) strain, submitted to the same treatment, showed no differences compared with untreated fish of the same strain (27). Similarly, both chronic alcohol exposure and alcohol withdrawal abolished social preference in WT AB strain zebrafish, but not in SF strain animals (53). Conversely, both alcohol exposure during embryonic development (64) and acute alcohol treatment (60, 65) were found to affect social preference in the two WT strains considered. Zebrafish social preference was also impaired when acute exposure to alchool was associated with taurine, a β-amino sulfonic acid with a neuromodulatory function that influences complex behaviors (21). Moreover, in AB larvae, chronic exposure to sodium valproate, an anti-epileptic drug, markedly reduced zebrafish social preference (45).

Reductions in zebrafish social preference were observed in sick adult fish with an inflammatory response induced by bacterin inoculation (17), and in adult zebrafish chronically exposed to untreated tannery effluents, this latter evidence indicating a neurotoxic action of bioaccumulated pollutants, predominantly heavy metals and toxic organic compounds (20). Chronic exposure to acesulfame, an artificial sweetener considered a new environmental pollutant, reduced social preference in adult WT AB strain fish (66).

Social preference in adult SF strain animals was not affected by administration of drugs such as lysergic acid diethylamide (54), ibogaine (58), or ketamine (55), whereas it has been found to be promoted by amphetamines (61) and decreased by administration of the psychotropic drugs fluoxetine and diazepam (62).

## Studies Carried Out With Genetically Engineered Zebrafish

This category includes studies investigating human neuropsychiatric disorders from a genetic point of view. Their main aim is to find a valid zebrafish model for autism spectrum disorder.

Recent studies have provided evidence suggesting that genetic differences result in different pattern of social behavior (74) since several genes participate in social preference development in zebrafish. A single mutation in the zebrafish gene *otpa*, involved in the development of “oxytocinergic” neurons and in hypothalamic functions, seemed to reduce fish social preference by impairing neuropeptide switching in the “oxytocin” neuronal system (67). Zebrafish *reln* mutants, characterized by alterations in the signaling pathway of reelin, a glycoprotein important for brain patterning during development, did not show reduced social preference (68). In zebrafish, knocking out of *dyrk1aa*, a gene that has been shown to exhibit features potentially relevant to human autism spectrum disorders, caused impairment of social preference (49). Instead, *oxtr* mutant fish, lacking a functional “oxytocin” receptor, displayed normal social preference for conspecifics (69).

## Discussion

The scientific literature suggests that the standardization of behavioral tests is an urgent requirement in many research fields (75, 76), since the implementation of standardized approaches to the study of social behavior in zebrafish will improve the reliability and usefulness of studies using these animals. Moreover, sharing methods and data between research laboratories could be the key to minimizing the number of animals used for the same purpose. Based on this evidence, the main goal of this review was to evaluate the determinants capable of influencing the results of social preference tests and to provide guidelines for standardizing social preference assays.

### Endogenous Factors

Several studies have found that zebrafish prefer socializing with conspecifics of similar phenotypes, age and size, and that these preferences are shown toward both live and computer-animated fish (19, 77, 78). On the contrary, Aslanzadeh et al. (49) suggested that zebrafish prefer to interact with fish of a larger size probably in order to reduce predation risks. With regard to zebrafish phenotypes, the studies considered in the present review focused mainly on two WT strains: AB and SF. Even though these strains look morphologically identical, the genetic variance between them could influence their social preferences (27). For this reason, and with a view to standardizing social preference assays, it is suggested that both size and phenotype should always be taken into account when performing social preference tests.

With regard to social stimuli, it is important, first, to mention the lack of data on the level of kinship of the subjects used to form the groups, even though it is conceivable that aggressive behaviors within groups could affect the preference of tested fish. The second point is the high variability of the number of individuals employed to test social stimuli. Indeed, the reviewed studies employed groups ranging from 1 to 15 adults, and from 1 to 6 larvae.

Moreover, in sexually mature zebrafish, a third crucial variable for interpretation of data in social stimuli studies is the sex ratio in the groups. After excluding the two studies carried out on zebrafish larvae, whose sex cannot be identified (45, 60), only a minority of the reviewed studies (4 of 26) were found to have used a balanced ratio of males to females as social stimuli. In four cases, the researchers employed only females in order to minimize male-to-male aggressive behavior during the test. Conversely, 17 studies did not clearly state the sex ratio of the fish utilized as the social stimulus, while one study employed a general mixed-sex group of fish (17). Since the social stimulus is a key variable when performing the social preference test, in accordance with Pham et al. (46), it is suggested to form stimulus groups with a balanced sex ratio, unless there are specific different goals.

Additionally, the sex of tested animals remains also a variability with 12 of the 26 studies testing an equal number of males and females, and eight using general mixed-sex samples. In four studies the authors did not clearly state the sex ratio of the tested fish, and in one study, not even the number of tested fish was reported (26).

Another aspect to be considered is the possible bias resulting from the personality (76) of the tested subject. In the studies reviewed—this review did not include the study by Seibt et al. (79), who tested five fish simultaneously—, only one individual was tested per experimental session. It could be crucial to measure, through standardized protocols, the boldness of fish before going on to test their social preference. This approach may make it possible to distinguish, and therefore determine, the sociability of both shyer and bolder individuals (80). Fontana et al. (21), for example, excluded from their analysis subjects that spent more than 80% of the interaction time immobile.

### Exogenous Factors

In relation to the possible role of environmental factors in social preference testing, the reviewed literature was found to lack specific data. The tank and chamber sizes, as well as the volume and temperature of the water and the brightness of the test room could all influence social behavior and should always be taken into account and reported in detail. The size and number of preference areas could also influence the results of the experiment and therefore give rise to biases when comparing different studies. In addition, as reported by Pham and colleagues, when performing a binary choice test with a single social stimulus, it is essential to alternate the left/right position of the social stimulus between trials to overcome any lateral bias in the fish (46).

Even though social preference is mediated by vibration and chemosensory cues (58), several studies have suggested that sociability in zebrafish is mainly dependent on vision (60, 81). On this basis, the morphological traits of the fish are the main factors to be taken into account when testing sociability, yet without neglecting the other sensory stimuli. Although Pham et al. (46) argue that the transparent dividers between the different test tank chambers should be as tightly sealed as possible to prevent any uncontrolled influence of chemosensory cues (46), the presence of non-visual stimuli could actually be essential in enabling subjects to recognize familiar or unfamiliar groups of fish (26). It has been shown that even zebrafish larvae can distinguish related conspecifics through olfactory signals (82). Thirteen of the 28 reviewed studies employed watertight chambers or separate tanks, and four studies used computer-animated images. Computer vision, robotics and virtual reality technologies, by allowing social stimuli to be repeated identically, could improve the consistency of the social preference test and minimize the number of animals used in the study, but this approach raises a number of important considerations, such as additional costs, the need for specific knowledge and expertise, and the complete absence of all sensory stimulation other than visual stimulation. Of the studies reviewed in the present investigation, only two definitely allowed olfactory stimulation during tests, while three studies used the modified T-maze, whose guillotine doors presumably allow the passage of some water. Conversely, six studies did not state clearly whether or not the barrier between the tested subject and the social stimulus was watertight.

Finally, when assessing social preference in zebrafish, the other main variables to consider are the duration of the habituation and interaction phases. In the reviewed studies, the habituation phase lasted from 30 s to 20 min, with a mode of 30 sec, but seven studies did not report the habituation time at all. The interaction phase lasted between 10 s and 30 min, with a mode of 5 min. Standardization of the test duration, unless there are special needs, would be beneficial, making it easier to compare different studies and to determine inter-assay variability. Like Raymond et al. (83), we suggest prolonging the habituation phase to 6 min, as a significant increase in exploratory behavior and a decrease in freezing behavior are typically observed in this time frame. As mentioned, the habituation phase could also be used to assess the personality of each tested individual before performing the social preference test.

### Recommendations

In compliance with the Reduction criterion, (84), and considering the findings of Angiulli et al. (52), we suggest that significant data may be obtained using a number of tested animals ranging from twelve to fifteen. Moreover, with regard to the social stimulus, the findings of the present review suggest using groups of four individuals per stimulus, well-matched for sex, development stage and familiarity.

The scientific literature on zebrafish social preference testing suggests that both endogenous and exogenous factors could influence the behavioral response of the observed subjects. Essentially, the endogenous factors are their morphological and behavioral characteristics, i.e., phenotype, while the exogenous factors include aspects of fish management, environmental parameters, and possible pharmacological treatments.

Considering the main objective of this investigation, we suggest that recommendations/guidelines for a standardized social preference test might be summarized as follows:

- Duration of habituation and interaction phase: 6 min. Assessing the time required by the tested fish to start displaying exploratory behavior could be useful for determining social preference in relation to animal personality.
- Social stimulus. The social stimulus group should comprise four conspecific fish, familiar with each other in order to prevent aggressive behaviors, and having a balanced sex ratio. These fish should be the same age/size as the tested fish.
- Apparatus. The apparatus used should be an adapted T-maze with non-watertight barriers, to allow social information to be derived also from chemosensory cues in situations in which kinship with the social group is crucial. On the contrary, the transparent dividers between the different test tank chambers should be as tightly sealed as possible when kinship with the social group may be a confounding variable.
- Environment. The water should be changed for each tested subject and the environmental conditions should be as similar as possible to those of the rearing tank. Changing the water can be a source of stress for these animals, and this sensitivity to changes in water conditions must be borne in mind when the dividers between the tank chambers are non-watertight. The volume and temperature of the water and the brightness of the test room should remain as constant as possible.

## Conclusion

Despite possible biases resulting from the impossibility of controlling all the variables involved, the social preference test remains a simple and versatile assay that could provide revealing new insights into the mechanisms of neuropsychiatric and neurodevelopmental diseases characterized by social ability impairments. Application of this test in neurobehavioral research might offer two main advantages. Compared to shoaling and schooling assays, social preference test has the advantage of allowing examination of a single individual as opposed to a group of individuals. Moreover, compared to the mirror biting test and the predator exposure test, social preference test might be considered a low-stress assay since the social stimulus is less threatening.

## Data Availability Statement

The original contributions generated in the study are included in the article/Supplementary Material, further inquiries can be directed to the corresponding author.

## Author Contributions

AO and RL: conceptualization, methodology, investigation, and resources and original draft preparation. FS, AO, RL, VN, and AG: writing, review, and editing. MM and BF: supervision. FS and AG: supervision and funding acquisition. FS: project administration. All authors contributed to the article and approved the submitted version.

## Conflict of Interest

The authors declare that the research was conducted in the absence of any commercial or financial relationships that could be construed as a potential conflict of interest.

## References

[1] Mayden RL Tang KL Conway KW Freyhof J Chamberlain S Haskins M Phylogenetic relationships of Danio within the order Cypriniformes: a framework for comparative and evolutionary studies of a model species. J ExpZool B Mol Dev Evol. (2007) 308B:642–54. 10.1002/jez.b.21175 17554749

[2] King A. Researchers Find Their Nemo. Cell. (2009) 139:843–6. 10.1016/j.cell.2009.11.019 19945369

[3] Suurväli J Whiteley AR Zheng Y Gharbi K Leptin M Wiehe T. The laboratory domestication of zebrafish: from diverse populations to inbred substrains. Mol Biol Evol. (2020) 37:1056–69. 10.1093/molbev/msz289 31808937 PMC7086173

[4] McClure MM McIntyre PB McCune AR Notes on the natural diet and habitat of eight danionin fishes, including the zebrafish Danio rerio. J Fish Biol. (2006) 69:553–70. 10.1111/j.1095-8649.2006.01125.x

[5] Spence R Fatema MK Reichard M Huq KA Wahab MA Ahmed ZF The distribution and habitat preferences of the zebrafish in Bangladesh. J Fish Biol. (2006) 69:1435–48. 10.1111/j.1095-8649.2006.01206.x

[6] Lawrence C The husbandry of zebrafish (*Danio rerio*): a review. Aquaculture. (2007) 269:1–20. 10.1016/j.aquaculture.2007.04.077

[7] van den Hurk R Schoonen W van Zoelen GA Lambert JGD. The biosynthesis of steroid glucuronides in the testis of the zebrafish, *Brachydanio rerio*, and their pheromonal function as ovulation inducers. Gen Comp Endocrinol. (1987) 68:179–88. 10.1016/0016-6480(87)90027-X 3428552

[8] Ribas L Piferrer F The zebrafish (*Danio rerio*) as a model organism, with emphasis on applications for finfish aquaculture research. Rev Aquaculture. (2014) 6:209–40. 10.1111/raq.12041

[9] Gerhard GS Kauffman EJ Wang X Stewart R Moore JL Kasales CJ. Life spans and senescent phenotypes in two strains of Zebrafish (*Danio rerio*). Exp Gerontol. (2002) 37:1055–68. 10.1016/S0531-5565(02)00088-8 12213556

[10] Lidster K Readman GD Prescott MJ Owen SF. International survey on the use and welfare of zebrafish *Danio rerio* in research. J Fish Biol. (2017) 90:1891–905. 10.1111/jfb.13278 28220489

[11] Aleström P D'Angelo L Midtlyng PJ Schorderet DF Schulte-Merker S Sohm F. Zebrafish: housing and husbandry recommendations. Lab Anim. (2020) 54:213–24. 10.1177/0023677219869037 31510859 PMC7301644

[12] Graham C von Keyserlingk MAG Franks B Zebrafish welfare: natural history, social motivation and behaviour. Appl Anim Behav Sci. (2018) 200:13–22. 10.1016/j.applanim.2017.11.005

[13] Suriyampola PS Shelton DS Shukla R Roy T Bhat A Martins EP. Zebrafish Social Behavior in the Wild. Zebrafish. (2016) 13:1–8. 10.1089/zeb.2015.1159 26671510

[14] Varga ZM. Aquaculture, husbandry, and shipping at the Zebrafish International Resource Center. Meth Cell Biol. (2016) 135:509–34. 10.1016/bs.mcb.2016.01.007 27443942

[15] Ellis T Yildiz HY López-Olmeda J Spedicato MT Tort L Øverli Ø. Cortisol and finfish welfare. Fish Physiol Biochem. (2012) 38:163–88. 10.1007/s10695-011-9568-y 22113503

[16] Fichi G Naef V Barca A Longo G Fronte B Verri T. Fishing in the cell powerhouse: zebrafish as a tool for exploration of mitochondrial defects affecting the nervous system. Int J Mol Sci. (2019) 20:2409. 10.3390/ijms20102409 31096646 PMC6567007

[17] Kirsten K Soares SM Koakoski G Carlos Kreutz L Barcellos LJG. Characterization of sickness behavior in zebrafish. Brain Behav Immun. (2018) 73:596–602. 10.1016/j.bbi.2018.07.004 29981831

[18] Naef V Mero S Fichi G D'Amore A Ogi A Gemignani F. Swimming in deep water: zebrafish modeling of complicated forms of hereditary spastic paraplegia and spastic ataxia. Front Neurosci. (2019) 13:1–17. 10.3389/fnins.2019.01311 31920481 PMC6914767

[19] Saverino C Gerlai R. The social zebrafish: behavioral responses to conspecific, heterospecific, and computer animated fish. Behav Brain Res. (2008) 191:77–87. 10.1016/j.bbr.2008.03.013 18423643 PMC2486438

[20] Chagas TQ da Silva Alvarez TG Montalvão MF Mesak C Rocha TL da Costa Araújo AP. Behavioral toxicity of tannery effluent in zebrafish (*Danio rerio*) used as model system. Sci Total Environ. (2019) 685:923–33. 10.1016/j.scitotenv.2019.06.253 31247439

[21] Fontana BD Stefanello FV Mezzomo NJ Müller TE Quadros VA Parker MO. Taurine modulates acute ethanol-induced social behavioral deficits and fear responses in adult zebrafish. J Psychiatr Res. (2018) 104:176–82. 10.1016/j.jpsychires.2018.08.008 30096615

[22] Stewart AM Braubach O Spitsbergen J Gerlai R Kalueff AV. Zebrafish models for translational neuroscience research: from tank to bedside. Trends Neurosci. (2014) 37:264–78. 10.1016/j.tins.2014.02.011 24726051 PMC4039217

[23] Rein B Ma K Yan Z. A standardized social preference protocol for measuring social deficits in mouse models of autism. Nat Protoc. (2020) 15:3464–77. 10.1038/s41596-020-0382-9 32895524 PMC8103520

[24] Beery AK Christensen JD Lee NS Blandino KL. Specificity in sociality: mice and prairie voles exhibit different patterns of peer affiliation. Front Behav Neurosci. (2018) 12:50. 10.3389/fnbeh.2018.00050 29615879 PMC5868120

[25] Geng Y Peterson RT. The zebrafish subcortical social brain as a model for studying social behavior disorders. Dis Models Mech. (2019) 12:dmm039446. 10.1242/dmm.039446 31413047 PMC6737945

[26] Norton WHJ Manceau L Reichmann F. The visually mediated social preference test: a novel technique to measure social behavior and behavioral disturbances in zebrafish. Methods Mol Biol. (2019) 2011:121–32. 10.1007/978-1-4939-9554-7_8 31273697

[27] Scerbina T Chatterjee D Gerlai R Dopamine receptor antagonism disrupts social preference in zebrafish: a strain comparison study. Amino Acids. (2012) 43:2059–72. 10.1007/s00726-012-1284-0 22491827 PMC3425734

[28] Pitcher TJ Parrish JK. Functions of shoaling behaviour in teleosts. In: Pitcher TJ editor. The Behaviour of Teleost Fishes. 2nd ed. London: Croom Helm. p. 363–439.

[29] Abaid N Porfiri M Fish in a ring: spatio-temporal pattern formation in one-dimensional animal groups. J R Soc Interface. (2010) 7:1441–53. 10.1098/rsif.2010.0175 20413559 PMC2935604

[30] Wakamatsu Y Ogino K Hirata H Swimming capability of zebrafish is governed by water temperature, caudal fin length and genetic background. Sci Rep. (2019) 9:16307 10.1038/s41598-019-52592-w 31704960 PMC6841939

[31] Bianco IH Kampff AR Engert F. Prey capture behavior evoked by simple visual stimuli in larval zebrafish. Front Syst Neurosci. (2011) 5:101. 10.3389/fnsys.2011.00101 22203793 PMC3240898

[32] Abreu MS Maximino C Banha F Anastácio PM Demin KA Kalueff AV. Emotional behavior in aquatic organisms? Lessons from crayfish and zebrafish. J Neurosci Res. (2020) 98:764–79. 10.1002/jnr.24550 31722127

[33] Oliveira RF. Mind the fish: zebrafish as a model in cognitive social neuroscience. Front Neural Circuits. (2013) 7:1–15. 10.3389/fncir.2013.00131 23964204 PMC3737460

[34] Miller N Gerlai R. From schooling to shoaling: patterns of collective motion in zebrafish (*Danio rerio*). PLoS ONE. (2012) 7:e48865. 10.1371/journal.pone.0048865 23166599 PMC3498229

[35] Darrow KO Harris WA. Characterization and Development of Courtship in Zebrafish, *Danio rerio*. Zebrafish. (2004) 1:40–5. 10.1089/154585404774101662 18248204

[36] Székely T Moore AJ Komdeur J Social Behaviour: Genes, Ecology and Evolution. Cambridge: Cambridge University Press (2010).

[37] Taborsky M Hofmann HA Beery AK Blumstein DT Hayes LD Lacey EA. Taxon matters: promoting integrative studies of social behavior. Trends Neurosci. (2015) 38:189–91. 10.1016/j.tins.2015.01.004 25656466

[38] Silverman JL Yang M Lord C Crawley JN Behavioural phenotyping assays for mouse models of autism. Nat Rev Neurosci. (2010) 11:490–502. 10.1038/nrn2851 20559336 PMC3087436

[39] Mahabir S Chatterjee D Buske C Gerlai R. Maturation of shoaling in two zebrafish strains: a behavioral and neurochemical analysis. Behav Brain Res. (2013) 247:1–8. 10.1016/j.bbr.2013.03.013 23518435 PMC3646909

[40] Robert G. Animated images in the analysis of zebrafish behavior. Curr Zool. (2017) 63:35–44. 10.1093/cz/zow077 29491961 PMC5804150

[41] Miller NY Gerlai R. Oscillations in shoal cohesion in zebrafish (*Danio rerio*). Behav Brain Res. (2008) 193:148–51. 10.1016/j.bbr.2008.05.004 18573546 PMC2709827

[42] Audira G Sampurna B Juniardi S Liang S-T Lai Y-H Hsiao C-D A versatile setup for measuring multiple behavior endpoints in zebrafish. Inventions. (2018) 3:75 10.3390/inventions3040075

[43] Spinello C Yang Y Macrì S Porfiri M Zebrafish adjust their behavior in response to an interactive robotic predator. Front Rob AI. (2019) 6:1–14. 10.3389/frobt.2019.00038 PMC7806020 33501054

[44] Robert G. Zebrafish antipredatory responses: a future for translational research? Behav Brain Res. (2010) 207:223–31. 10.1016/j.bbr.2009.10.008 19836422 PMC3203216

[45] Liu X Zhang Y Lin J Xia Q Guo N Li Q. Social preference deficits in juvenile zebrafish induced by early chronic exposure to sodium valproate. Front Behav Neurosci. (2016) 10:1–8. 10.3389/fnbeh.2016.00201 27812327 PMC5071328

[46] Pham M Raymond J Hester J Kyzar E Gaikwad S Bruce I Assessing social behavior phenotypes in adult zebrafish: shoaling, social preference, and mirror biting tests. In: Kalueff AV Stewar AM editors. Zebrafish Protocols for Neurobehavioral Research. Berlin: Springer (2012). p. 231–46.

[47] Stowers JR Hofbauer M Bastien R Griessner J Higgins P Farooqui S. Virtual reality for freely moving animals. Nat Methods. (2017) 14:995–1002. 10.1038/nmeth.4399 28825703 PMC6485657

[48] Deacon RMJ Rawlins JNP. T-maze alternation in the rodent. Nat Protoc. (2006) 1:7–12. 10.1038/nprot.2006.2 17406205

[49] Aslanzadeh M Ariyasiri K Kim O-H Choi T-I Lim J-H Kim H-G. The body size of stimulus conspecifics affects social preference in a binary choice task in wild-type, but not in dyrk1aa mutant, Zebrafish. Zebrafish. (2019) 16:262–7. 10.1089/zeb.2018.1717 31058587

[50] Landin J Hovey D Xu B Lagman D Zettergren A Larhammar D. Oxytocin receptors regulate social preference in zebrafish. Sci Rep. (2020) 10:5435. 10.1038/s41598-020-61073-4 32214126 PMC7096398

[51] Engeszer RE Ryan MJ Parichy DM. Learned social preference in zebrafish. Curr Biol. (2004) 14:881–4. 10.1016/j.cub.2004.04.042 15186744

[52] Angiulli E Pagliara V Cioni C Frabetti F Pizzetti F Alleva E. Increase in environmental temperature affects exploratory behaviour, anxiety and social preference in Danio rerio. Sci Rep. (2020) 10:5385. 10.1038/s41598-020-62331-1 32214187 PMC7096496

[53] Gerlai R Chatterjee D Pereira T Sawashima T Krishnannair R. Acute and chronic alcohol dose: population differences in behavior and neurochemistry of zebrafish. Genes Brain Behav. (2009) 8:586–99. 10.1111/j.1601-183X.2009.00488.x 19243447 PMC2880629

[54] Grossman L Utterback E Stewart A Gaikwad S Chung KM Suciu C. Characterization of behavioral and endocrine effects of LSD on zebrafish. Behav Brain Res. (2010) 214:277–84. 10.1016/j.bbr.2010.05.039 20561961

[55] Riehl R Kyzar E Allain A Green J Hook M Monnig L. Behavioral and physiological effects of acute ketamine exposure in adult zebrafish. Neurotoxicol Teratol. (2011) 33:658–67. 10.1016/j.ntt.2011.05.011 21683787

[56] Sison M Gerlai R. Behavioral performance altering effects of MK-801 in zebrafish (*Danio rerio*). Behav Brain Res. (2011) 220:331–7. 10.1016/j.bbr.2011.02.019 21333690 PMC3072452

[57] Braida D Donzelli A Martucci R Capurro V Busnelli M Chini B. Neurohypophyseal hormones manipulation modulate social and anxiety-related behavior in zebrafish. Psychopharmacology. (2012) 220:319–30. 10.1007/s00213-011-2482-2 21956239

[58] Cachat J Kyzar EJ Collins C Gaikwad S Green J Roth A. Unique and potent effects of acute ibogaine on zebrafish: the developing utility of novel aquatic models for hallucinogenic drug research. Behav Brain Res. (2013) 236:258–69. 10.1016/j.bbr.2012.08.041 22974549

[59] Lindeyer CM Langen EMA Swaney WT Reader SM Nonapeptide influences on social behaviour: effects of vasotocin and isotocin on shoaling and interaction in zebrafish. Behaviour. (2015) 152:897–915. 10.1163/1568539X-00003261

[60] Dreosti E Lopes G Kampff AR Wilson SW Development of social behavior in young zebrafish. Front Neural Circuits. (2015) 9:1–9. 10.3389/fncir.2015.00039 26347614 PMC4539524

[61] Ponzoni L Sala M Braida D. Ritanserin-sensitive receptors modulate the prosocial and the anxiolytic effect of MDMA derivatives, DOB and PMA, in zebrafish. Behav Brain Res. (2016) 314:181–9. 10.1016/j.bbr.2016.08.009 27506653

[62] Giacomini ACVV Abreu MS Giacomini LV Siebel AM Zimerman FF Rambo CL. Fluoxetine and diazepam acutely modulate stress induced-behavior. Behav Brain Res. (2016) 296:301–10. 10.1016/j.bbr.2015.09.027 26403161

[63] Zimmermann FF Gaspary KV Siebel AM Bonan CD. Oxytocin reversed MK-801-induced social interaction and aggression deficits in zebrafish. Behav Brain Res. (2016) 311:368–74. 10.1016/j.bbr.2016.05.059 27247142

[64] Baggio S Mussulini BH de Oliveira DL Gerlai R Rico EP. Embryonic alcohol exposure leading to social avoidance and altered anxiety responses in adult zebrafish. Behav Brain Res. (2018) 352:62–9. 10.1016/j.bbr.2017.08.039 28882694

[65] Paiva IM Sartori BM Castro TFD Lunkes LC Virote B do CR. Behavioral plasticity and gene regulation in the brain during an intermittent ethanol exposure in adult zebrafish population. Pharmacology Biochemistry and Behavior, 192, 172909. 10.1016/j.pbb.2020.172909 32194086

[66] Dong G Li X Han G Du L Li M. Zebrafish neuro-behavioral profiles altered by acesulfame (ACE) within the range of “no observed effect concentrations (NOECs).” Chemosphere. (2020) 243:125431. 10.1016/j.chemosphere.2019.125431 31995882

[67] Wircer E Blechman J Borodovsky N Tsoory M Nunes AR Oliveira RF. Homeodomain protein Otp affects developmental neuropeptide switching in oxytocin neurons associated with a long-term effect on social behavior. ELife. (2017) 6:1–25. 10.7554/eLife.22170 28094761 PMC5293488

[68] Dalla Vecchia E Di Donato V Young AMJ Del Bene F Norton WHJ. Reelin signaling controls the preference for social novelty in zebrafish. Front Behav Neurosci. (2019) 13:1–16. 10.3389/fnbeh.2019.00214 31607872 PMC6761276

[69] Ribeiro D Nunes AR Gliksberg M Anbalagan S Levkowitz G Oliveira RF Oxytocin receptor signalling modulates novelty recognition but not social preference in zebrafish. J Neuroendocrinol. (2020) 2019:1–9. 10.1111/jne.12834 31961994

[70] White RM Sessa A Burke C Bowman T LeBlanc J Ceol C. Transparent adult zebrafish as a tool for *in vivo* transplantation analysis. Cell Stem Cell. (2008) 2:183–9. 10.1016/j.stem.2007.11.002 18371439 PMC2292119

[71] Reddon AR O'Connor CM Marsh-Rollo SE Balshine S Effects of isotocin on social responses in a cooperatively breeding fish. Anim Behav. (2012) 84:753–60. 10.1016/j.anbehav.2012.07.021

[72] Ogi A Mariti C Baragli P Sergi V Gazzano A. Effects of stroking on salivary oxytocin and cortisol in guide dogs: preliminary results. Animals. (2020) 10:708. 10.3390/ani10040708 32325673 PMC7222818

[73] Rincon AV Deschner T Schülke O Ostner J. Oxytocin increases after affiliative interactions in male Barbary macaques. Horm Behav. (2020) 119:104661. 10.1016/j.yhbeh.2019.104661 31883945

[74] Tang W Davidson JD Zhang G Conen KE Fang J Serluca F. Genetic control of collective behavior in zebrafish. IScience. (2020) 23:100942. 10.1016/j.isci.2020.100942 32179471 PMC7068127

[75] Wahlsten D. Standardizing tests of mouse behavior: reasons, recommendations, and reality. Physiol Behav. (2001) 73:695–704. 10.1016/S0031-9384(01)00527-3 11566204

[76] Way GP Kiesel AL Ruhl N Snekser JL McRobert SP. Sex differences in a shoaling-boldness behavioral syndrome, but no link with aggression. Behav Processes. (2015) 113:7–12. 10.1016/j.beproc.2014.12.014 25562194

[77] Rosenthal GG Ryan MJ Assortative preferences for stripes in danios. Anim Behav. (2005) 70:1063–6. 10.1016/j.anbehav.2005.02.005

[78] Snekser J Ruhl N Bauer K McRobert S The influence of sex and phenotype on shoaling decisions in zebraf. Int J Comp Psychol. (2010) 23:70–81.

[79] Seibt KJ Piato AL da Luz Oliveira R Capiotti KM Vianna MR Bonan CD Antipsychotic drugs reverse MK-801-induced cognitive and social interaction deficits in zebrafish (*Danio rerio*). Behav Brain Res. (2011) 224:135–9. 10.1016/j.bbr.2011.05.034 21669233

[80] Way GP Southwell M McRobert SP. Boldness, aggression, and shoaling assays for zebrafish behavioral syndromes. J Visualized Exp. (2016) 2016:1–8. 10.3791/54049 27684060 PMC5091962

[81] Engeszer RE Wang G Ryan MJ Parichy DM. Sex-specific perceptual spaces for a vertebrate basal social aggregative behavior. Proc Natl Acad Sci. (2008) 105:929–33. 10.1073/pnas.0708778105 18199839 PMC2242707

[82] Gerlach G Hodgins-Davis A Avolio C Schunter C. Kin recognition in zebrafish: a 24-hour window for olfactory imprinting. Proc R Soc B: Biol Sci. (2008) 275:2165–70. 10.1098/rspb.2008.0647 18544507 PMC2603219

[83] Raymond J Chanin S Stewart AM Kyzar E Gaikwad S Roth A Assessing habituation phenotypes in adult zebrafish: intra-and inter-trial habituation in the novel tank test. In: Kalueff AV Stewar AM editors. Zebrafish Protocols for Neurobehavioral Research. Totowa, NJ: Humana Press (2012). p. 273–85.

[84] National Research Council (US), Committee on Recognition and Alleviation of Pain in Laboratory Animals *Pain in research* animals: general principles and considerations. In: Recognition and Alleviation of Pain in Laboratory Animals. Washington, DC: National Academies Press (2009). 20662126

