# Supplementary figures and images for: Social Preference Tests in Zebrafish: A Systematic Review
Source: Front Vet Sci. 2021 Jan 22;7:590057. doi: 10.3389/fvets.2020.590057 (PMC7862119; doi:10.3389/fvets.2020.590057)

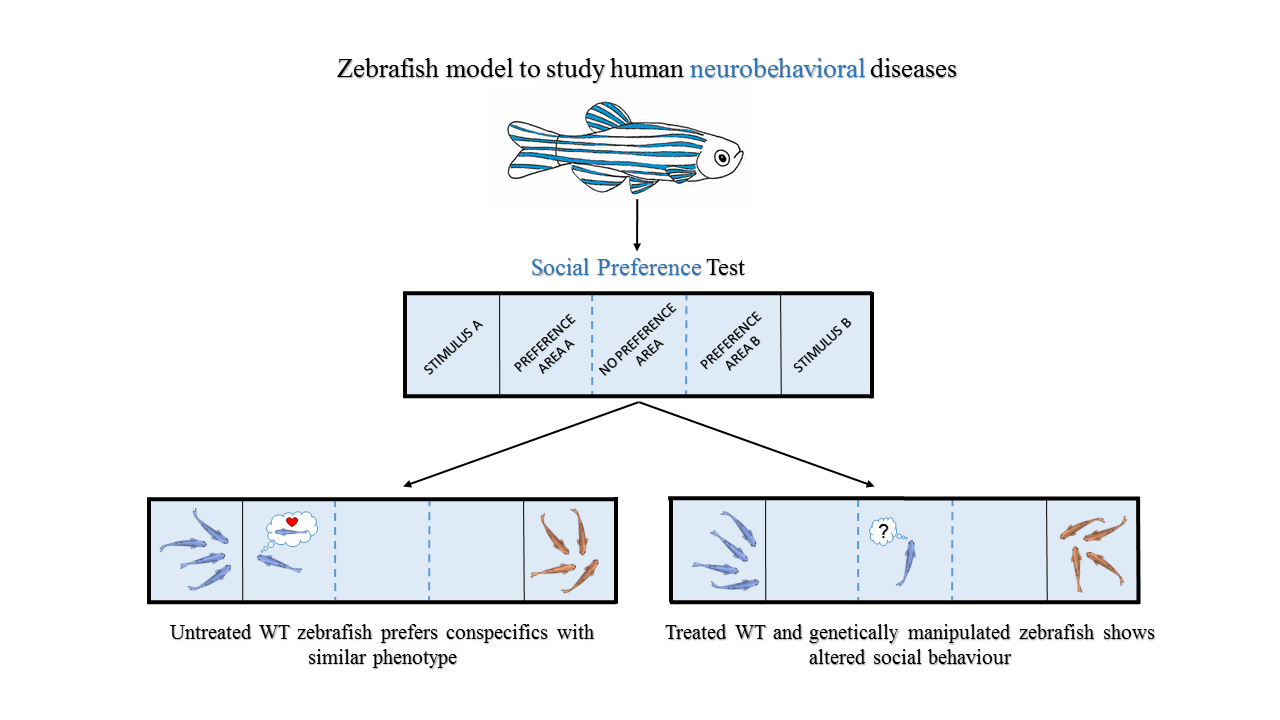

Supplement: Supplementary file 2 [file Image_1.TIF]
